# Detection of *Borrelia burgdorferi* antigens in tissues and plasma during early infection in a mouse model

**DOI:** 10.1038/s41598-021-96861-z

**Published:** 2021-08-30

**Authors:** Victoria Dolange, Stéphanie Simon, Nathalie Morel

**Affiliations:** Paris-Saclay University, CEA, INRAE, Medicines and Healthcare Technologies Department (DMTS), SPI, 91191 Gif-sur-Yvette, France

**Keywords:** Diagnostic markers, Infectious diseases

## Abstract

*Borrelia burgdorferi* is the causative agent of Lyme borreliosis, which is the most common tick-borne human disease in Europe and North America. Currently, the diagnosis of Lyme borreliosis is based on serological tests allowing indirect detection of anti-*Borrelia* antibodies produced by patients. Their main drawback is a lack of sensitivity in the early phase of disease and an incapacity to prove an active infection. Direct diagnostic tests are clearly needed. The objectives of this study were to produce tools allowing sensitive detection of potential circulating *Borrelia* antigens and to evaluate them in a mouse model. We focused on two potential early bacterial makers, the highly variable OspC protein and the conserved protein FlaB. High-affinity monoclonal antibodies were produced and used to establish various immunoassays and western blot detection. A very good limit of detection for OspC as low as 17 pg/mL of sample was achieved with SPIE-IA. In infected mice, we were able to measure OspC in plasma with a mean value of 10 ng/mL at 7 days post-inoculation. This result suggests that OspC could be a good blood marker for diagnosis of Lyme borreliosis and that the tools developed during this study could be very useful.

## Introduction

Lyme borreliosis is the most common reported tick-borne infectious disease in Europe^1^ and North America^2^. This infection is caused by spirochetes of *Borrelia burgdorferi* sensu lato (s.l.) of which at least five species (*B. burgdorferi* sensu stricto, *B. afzelii*, *B. garinii, B. bavariensis* and *B. spielmanii*) are pathogenic to humans^3^. The clinical manifestations are varied and polymorphic (cutaneous, neurological and rheumatological)^4^. The early infection phase is characterized by typical erythema migrans (EM) in 70–80% of cases^5^, often associated with nonspecific symptoms such as fever, myalgia, fatigue and joint pain^6^. If patients are not treated, the disease may progress to an early disseminated stage by hematogenous dissemination of the bacteria toward different organs and skin^7^. During this phase multiple cutaneous signs and cardiac, neurological and joint symptoms can occur. Late manifestations may occur several months to years after the onset of the infection and include mainly arthritis, acrodermatitis chronica atrophicans and late neuroborreliosis^8^.

In the absence of typical EM, the diagnosis of Lyme disease is difficult to establish due to the complexity of the clinical diagnosis^3,9^ and insufficient performances of the related laboratory assays. If there is a suspected bite and clinical signs suggestive of Lyme disease, serological tests based on detection of the host antibody response against *Borrelia* can be done^10^. The recommended approach for laboratory diagnosis of Lyme borreliosis is the two-tiered testing strategy, which includes an indirect enzyme-linked immunosorbent assay (ELISA) followed by a more specific western blot in order to rule out false positives^11^. These tests do not prove the existence of an active infection and are not recommended in the early localized phase of the disease. A recent meta-analysis showed that serological tests have heterogeneous sensitivity depending on the stage of the disease with only 50% during early manifestations (3–30 days post-infection)^12,13^. At this stage, the low sensitivity of serological testing is due to a weak or absent antibody response. Another pitfall of serological testing is the lack of standardization of the assay, which uses various recombinant proteins or whole-cell heterogeneous *Borrelia* to capture specific antibodies, thus producing undesirable variations in the test results. Direct detection of *Borrelia* in patients is difficult. Culture is not routinely used due to low sensitivity and a long incubation period of up to 12 weeks for bacterial growth^14^. Polymerase chain reaction (PCR) is generally very efficient in pathogen detection^15^, but in the case of Lyme disease the sensitivity is insufficient in blood due to the very low level of circulating bacteria^14^. PCR in synovial biopsy ranges in sensitivity from 40 to 90% and can be useful for diagnosis of Lyme arthritis. However*, Borrelia* DNA may remain in tissues after treatment and so is not a proof of active infection^16,17^.

Reliable, accurate, sensitive and rapid tests for active infection are thus needed for better care management of patients as soon as possible before they develop disseminated Lyme disease. Few studies have reported the direct detection of *Borrelia* protein in patients. In the past, assays based on capture and detection of Lyme antigen in urine or blood using anti-*Borrelia* polyclonal antibodies were developed^18^ but were later invalidated for measurement in patients due to low accuracy, sensitivity and specificity^19^. More recently, a new method based on the concentration of outer surface protein A (OspA) in a large volume of urine followed by detection with western blot was reported to be efficient in the early detection of active infection^20^, but robust validation is still needed.

The aim of the present work was to develop specific and sensitive immunoassays based on the direct detection of bacterial antigens. This first involves the production of a well-characterized and high-affinity monoclonal antibodies (mAbs) against potential circulating *Borrelia* antigens and, second, the development of sensitive immunological methods that could be useful for direct diagnosis of Lyme disease. We focused on two *Borrelia* proteins, flagellin B (FlaB), a major component of the periplasmic flagellar filament crucial for bacterial mobility, and OspC, a lipoprotein needed for the establishment of early localized infection. Both proteins are described as early immunodominant antigens after infection^21^ and were also shown to be detectable in skin biopsy from humans with EM lesions using mass spectrometry analysis^22^. Two strategies were pursued for immunization. For FlaB, which is a highly conserved protein among *Borrelia* species, we used a recombinant protein as immunogen. For OspC, considering its high variability between *B. burgdorferi* species and strains of the same species, an approach using a highly conserved OspC peptide motif as immunogen was investigated. After characterization and selection of the best mAbs, we developed two sensitive detection methods. One is based on antigen immunocapture followed by western blot detection, while the second uses solid phase immobilized epitope immunoassays (SPIE-IA)^23^. These assays were evaluated for their capacity to detect FlaB and OspC in the tissues and plasma of experimentally infected mice. This study shows that OspC could be a good blood marker, for direct early diagnosis of Lyme disease.

## Results

### Production and characterization of monoclonal antibodies directed against FlaB protein

Given that FlaB protein has a high sequence homology between different *Borrelia* strains and species, we raised antibodies against a recombinant FlaB expressed in *E. coli*. Throughout the screening and cloning process, secreted hybridoma antibodies were assayed using two methods. The first measures antibody binding with biotinylated recombinant FlaB and the second measures binding with whole-cell *Borrelia burgdorferi* extract (strain BO23) coated on a solid phase. A total of 21 hybridomas were selected for their high reactivity for both sources of FlaB and were successfully cloned and stabilized. Purified mAbs were further characterized by sandwich immunoassays, western blots and affinity determination by biolayer interferometry.

In an attempt to develop a sensitive ELISA for FlaB detection, we performed an mAb combinatorial analysis as described in materials and methods. Among the 441 combinations resulting from the 21 anti-FlaB mAbs, 361 pairs of antibodies provided good detection for 10^5^ *Borrelia*. The best sandwich immunoassays, including either FlaB-15 or FlaB-18 as capture mAb and FlaB-20 as tracer, were further evaluated in serial dilutions using an extract of *Borrelia* (data not shown). The limit of detection (LoD) was around 1 × 10^4^ *Borrelia* per mL.

**Figure 1:**
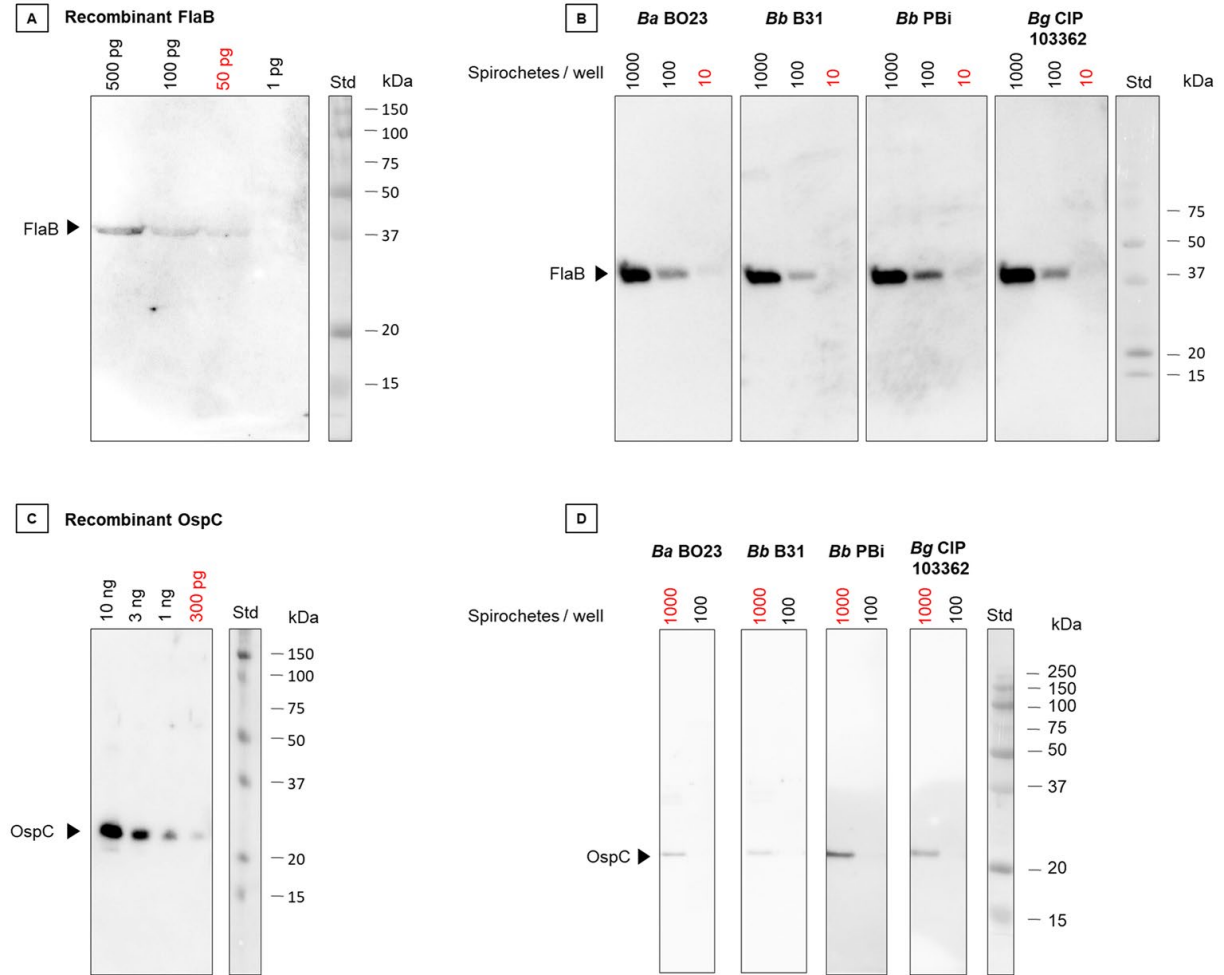
Western blot detection of FlaB and OspC using the best selected mAb. (**A**) Range of recombinant FlaB from 500 to 1 pg per well detected with FlaB-15 mAb and (**C**) recombinant OspC from 10 ng to 40 pg per well detected with C10-11 mAb. (**B**, **D**) Various *Borrelia* strains loaded at 10^3^ and 10^2^ bacteria per lane and detected with FlaB-15 (**B**) and C10-11 mAbs (**D**). Std: Molecular weight markers and protein sizes (kDa) are indicated on the right side of the gels. Uncropped original images are reported in Fig. S2.

The 21 anti-FlaB mAbs were tested for their ability to recognize recombinant FlaB and whole-cell *Borrelia* by western blot*.* A single band around 41 kDa, corresponding to the molecular mass of FlaB, was observed with all 21 mAbs, with recombinant FlaB and *Borrelia* cells. Three highly efficient mAbs, FlaB-15, FlaB-18 and FlaB-20, were further evaluated on a range of bacteria from 10 to 1000 spirochetes and recombinant FlaB. As shown in Fig. 1A,B, the most efficient anti-FlaB mAb, FlaB-15, allows highly sensitive detection of different species of *Borrelia burgdorferi* (*B. burgdorferi, B. garinii and B. afzelii*) with 10–100 spirochetes detected according to the strain and 50 pg of recombinant FlaB.

Kinetic affinity parameters of the best mAbs, previously identified by ELISA and western blot, were determined by biolayer interferometry using recombinant FlaB. All mAbs presented quite similar kinetic constants and a high affinity for FlaB, with K_D_ between 0.23 and 2.10 nM (see Table 1), and are thus potentially suitable for sensitive detection of FlaB. However, kinetic constants remain purely indicative because experiments were not performed with native FlaB and we cannot ascertain whether recombinant FlaB produced in inclusion bodies of *E. coli* and refolded in vitro has the same conformation and solvent exposure as native polymerized FlaB in the flagella.

**Table 1:** Kinetic parameters of mAbs for flagellin B and OspC proteins.

Antigen	Antibody	k_a_ (/M/s) × 10^5a^	k_dis_ (/s) × 10^–4a^	K_D_ (M) × 10^–9a^
FlaB	FlaB-15	1.03 ± 0.001	0.24 ± 0.008	0.23 ± 0.007
	FlaB-18	2.05 ± 0.006	0.90 ± 0.021	0.44 ± 0.010
	FlaB-20	1.09 ± 0.003	2.28 ± 0.023	2.10 ± 0.022
OspC	C10-11	1,99 ± 0.003	0.92 ± 0.009	0.46 ± 0.004
	C10-13	2.03 ± 0.005	3.15 ± 0.015	1.55 ± 0.008
	C10-14	1.39 ± 0.003	2.47 ± 0.013	1,78 ± 0.01
	C10-16	1.79 ± 0.005	3.29 ± 0.015	1.84 ± 0.010
	C10-17	1.81 ± 0.005	3.11 ± 0.016	1,72 ± 0.010
	C10-19	1.72 ± 0.006	4.04 ± 0.017	2.36 ± 0.013
	C10-20	1.31 ± 0.003	2.67 ± 0.028	2.02 ± 0.021

Mean and standard deviation values of equilibrium dissociation constant (K_D_), association rate (k_a_) and dissociation rate (k_dis_) were extracted from the curve-fitting analysis of mAb association and dissociation successive dilutions (out of 7).

^a^Values determined by the Octet Data Analysis HT 10.0 software using a 1:1 binding model, and expressed as mean ± SEM.

### Production of monoclonal antibodies against a conserved OspC peptide sequence

OspC amino acid sequence varies greatly between different strains of *B. burgdorferi* s.l. The search for conserved immunogenic epitopes of OspC allowed identification of a highly conserved sequence corresponding to the C-terminal 10-amino-acid peptide of OspC, known as the C10 peptide^24^. The sequence consensus of this peptide found in the great majority of pathogenic *Borrelia* is PVVAESPKKP^24,25^. Comparison of 89 non-redundant OspC amino acid sequences of *Borrelia burgdorferi* s.l. in the NCBI database shows that this peptide was 100% identical in 69 sequences, i.e. more than 80% of the strains, whereas the other sequences showed only slight variations of 1 or 2 amino acids. Thus, by using the C10 consensus peptide as immunogen, we assumed that we were targeting the OspC protein of the major infectious strains.

Mice were immunized with BSA coupled to C10 peptide through a cysteine added to its N-terminal extremity. After fusion of spleen cells with myeloma cells, hybridomas were screened using three different assays based on the binding of antibodies to the C10 peptide, to full-length recombinant OspC and to whole-cell *Borrelia* extract (strain BO23). Of 189 hybridomas multiplying after fusion, a total of 7 hybridomas satisfying all screening assays were finally cloned and stabilized.

Kinetic affinity parameters of the anti-C10 peptide mAbs are reported in Table 1. All mAbs were able to bind OspC with a strong affinity in the low nanomolar range, suggesting that the C-terminal epitope C10 is fully exposed in the recombinant OspC protein. The C10-11 mAb has the highest affinity for OspC, with a K_D_ of 0.46 nM, due essentially to its very low dissociation constant.

### Competition experiments with C10 peptide variants

**Figure 2:**
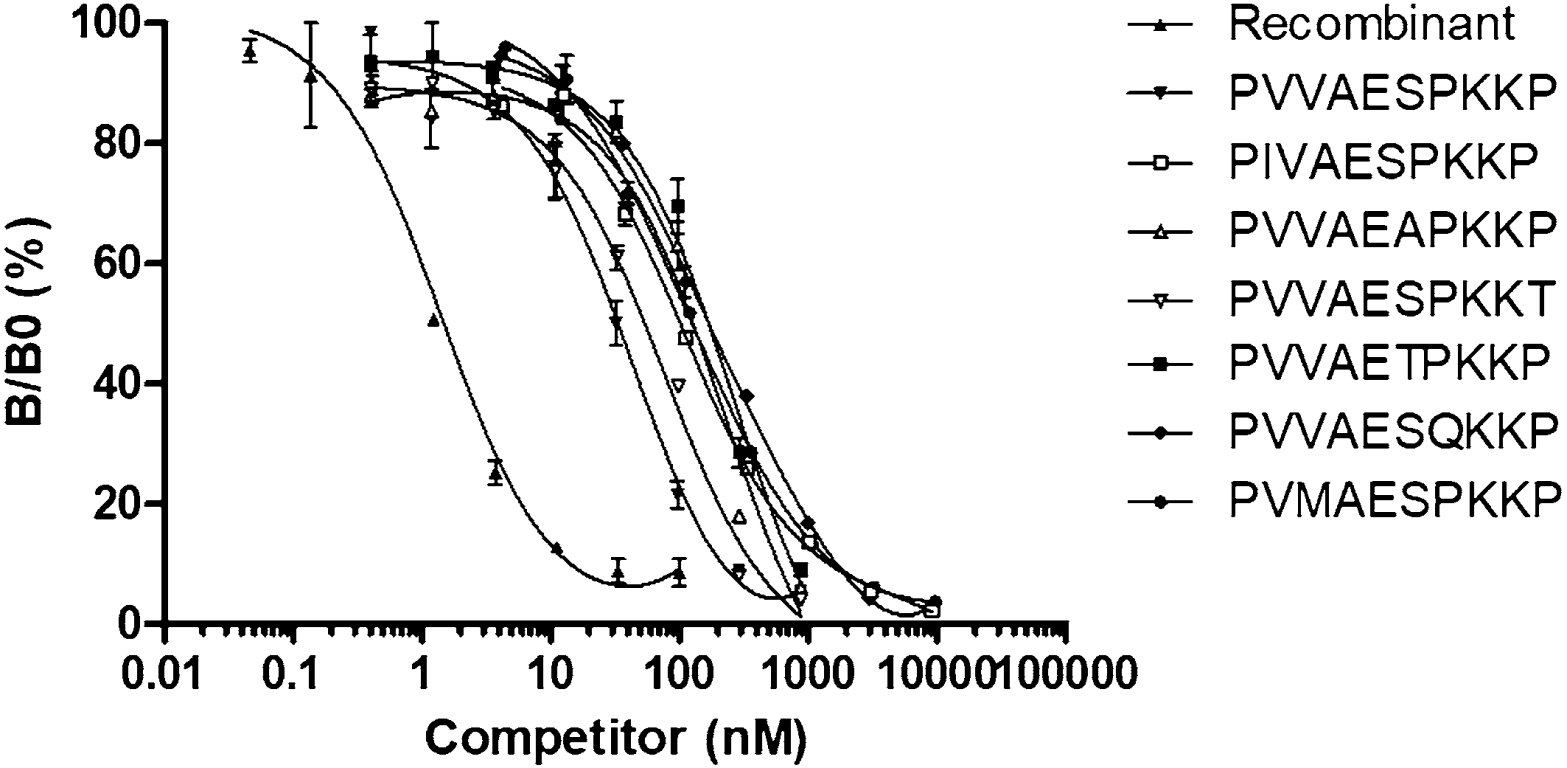
Competition ELISA experiments with various C10 peptide variants. B/B0 represents the percentage of binding of the C10-11 mAb with the C10 peptide tracer. The results are expressed in terms of B/B0 as a function of the logarithm of the competing C10 variant or OspC. Data shown are representative of three independent experiments. Data from non-competing peptides (peptides 4; 6; 8 and 11 in Table 2) are not included in the graph.

In order to evaluate the capacity of the C10-11 mAb to bind OspC protein from all potential pathogenic *Borrelia burgdorferi* s.l., competition experiments were performed with various peptide sequences corresponding to the C-terminal OspC decapeptide of *Borrelia burgdorferi* s.l. in the NCBI database. From 89 sequences of OspC, we identified ten different decapeptides presenting one or two amino acid substitutions with regard to the C10 consensus sequence (see Table 2). For the competition experiments, various concentrations of each peptide were mixed with the acetylcholinesterase (AChE)-labeled C10 peptide and added to C10-11 mAb-coated plates. The apparent affinity (K_app_) of the C10-11 mAb for each peptide variant was determined as the concentration of peptide inducing 50% inhibition of the binding of labeled C10 peptide. For four peptides (peptides 4, 6, 8, and 11), no inhibition was observed, while for three of them there is only one amino acid substitution. The same result was observed with all other anti-C10 peptide mAbs produced. As shown in Fig. 2, the six other peptides were able to inhibit the binding of C10 peptide, allowing determination of a K_app_ ranging from 60 to 179 nM, i.e. 2–5 times greater than the K_app_ of the reference C10 peptide (33 nM). These results suggest that the OspC variants corresponding to these peptides should be recognized by the C10-11 mAb, but with a lower affinity resulting in a lower sensitivity of detection. For recombinant OspC, a K_app_ of 1 nM was determined, which is consistent with the data obtained by biolayer interferometry. The discrepancy in affinity between the C10 peptide and the OspC protein may possibly reflect a conformational difference between the free C10 peptide and the same peptide engaged in a peptide bond with the rest of OspC protein, as was the immunogen. According to the frequency of each variant reported in the NCBI database, the mAb C10-11 could thus detect OspC protein of more than 90% of *Borrelia burgdorferi* strains.

**Table 2:** Variant of C10 C-terminal peptide. Apparent dissociation constant (K_app_) of C10-11 mAb for C10 peptide variants was calculated based on the fitting performed in the competition experiments (Fig. 1). The representativeness of the different sequences of the C10 peptide among *Borrelia burgdorferi* sl. is given in percentages. NB means no binding. *BBss: Borrelia burgdorferi sensu stricto, Ba: Borrelia afzelii, Bg: B. garinii, Bbav: B. bavariensis*.

C-terminal peptide	K_app_ (nM)	Representativeness (%)	Example of *B. burgdorferi* s.l. strains
1 : P V V A E S P K K P	33	80.9	*Bb*ss B31; Bbss Bre13; Bbss ACA1; Bbss PLe; Bb PBo; *Ba* BO23; Ba PKo; Ba HT25; Bg PLi; Bg HB19; Bg PBes
2 : P V V A E A P K K P	146	0.9	*Bg* PBr
3 : P V V A E S P K K T	60	0.9	*Bbss* PWag
4 : P V V A E S P K N P	NB	0.9	*Bbss* JD1, Ba K78
5 : P V V A E T P K K P	179	5.5	*Bbav* PBi
6 : P V V A E N P K K P	NB	0.9	*Bbss* F045
7 : P V V A E S Q K K P	170	0.9	*Ba* BaC22
8 : P V V V E S P K K P	NB	1.8	*Bbss* ZS7
9 : P V M A E S P K K P	125	0.9	*Bg* 935 T
10 : P I V A E S P K K P	100	3.6	*Bbss* N40; *Bbss* LDP74
11 : P I V A E S P K N P	NB	2.7	*Bbss* 297

### Development of a solid-phase immobilized epitope immunoassay (SPIE-IA) for OspC protein

**Figure 3:**
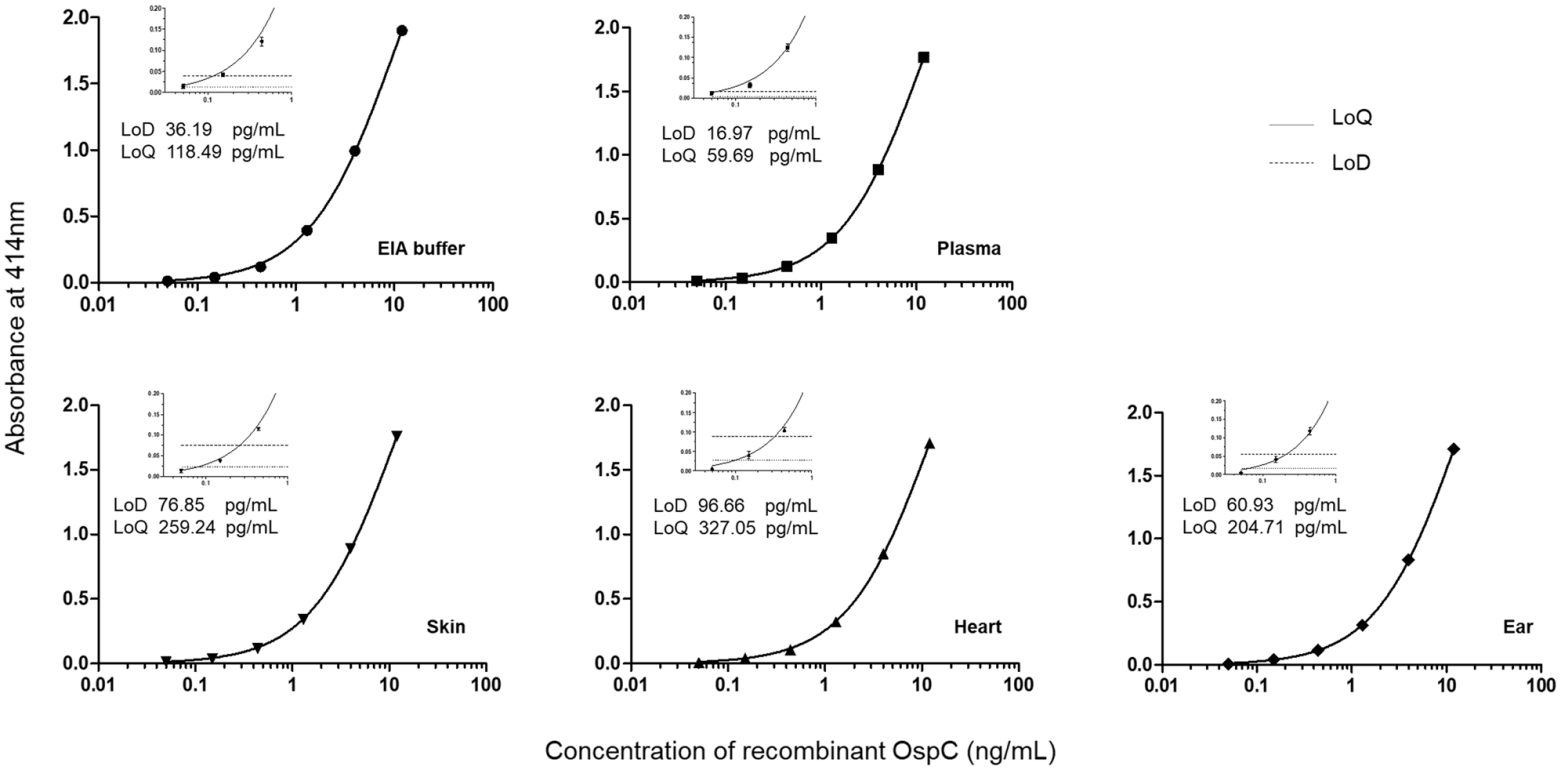
Standards curves obtained with solid phase-immobilized epitope immunoassay (SPIE-IA) in various complex matrices. Serial dilutions of OspC protein were performed in EIA buffer and in different mouse tissues or fluid. The theoretical limit of detection (LoD) and limit of quantification (LoQ) are indicated for each tissue. Thin dotted and dashed horizontal lines represent absorbance reached for the detection limit (three standard deviations) and quantification limit (10 standard deviations), respectively. Data points represent mean values obtained from three independent experiments each including a duplicate and error bars represent standard deviations (n = 6). The nonspecific binding was subtracted from data.

SPIE-IA technology, developed several years ago in our laboratory, is the most appropriate method for measurement of small molecules bearing a single epitope in excess reagent format^26^. This method is based on the use of a single epitope, such as the C10 peptide, recognized successively by a capture antibody and a tracer antibody (see “Methods”). To identify the best pair of antibodies for OspC detection by SPIE-IA, antibody combinatorial analysis was performed as described in materials and methods. The best combination, involving C10-11 as well as capture and tracer antibody, was selected according to the strongest signal-to-noise ratio observed with OspC protein. A standard curve established with OspC ranging from 50 pg/mL to 12 ng/mL and fitted using a nonlinear regression model is presented in Fig. 3. An LoD of 36 pg/mL was determined in assay buffer. Because compounds in biological matrixes may affect the specific binding between antibodies and targets possibly resulting in decreased sensitivity and specificity of immunoassays, we evaluated the matrix effect of different mouse tissues or fluid on the detection of recombinant OspC. The samples were prepared as tenfold diluted extract of tissues (5% W/V) or plasma. As shown in Fig. 3, taking the results obtained for buffer as reference, few differences were observed in sample matrices. The LoD and limit of quantification (LoQ) varied as a function of the nonspecific binding (subtracted in the curves shown), with a loss of sensitivity when nonspecific binding increased. The higher matrix effect was observed in heart samples for which the LoD was reduced by a factor of 2.5, whereas in plasma LoD was increased by a factor 2. These results show that C10 peptide SPIE-IA could be used to detect OspC in various tissues and plasma with a sensitivity as low as 17 pg/mL of sample (approximately 85 attomol by assay).

### Evaluation of anti-C10 peptide for OspC western blot detection

A first screening of the 7 mAbs was performed on the recombinant OspC protein at 5 µg/mL and against *B. garinii*, which strongly expresses OspC in vitro as checked by SDS-PAGE. All mAbs recognized the recombinant OspC and the native OspC of *Borrelia garinii* with an apparent molecular weight around 23 kDa. The more efficient antibody, mAb C10-11, was further evaluated on four different *Borrelia* strains that express OspC in culture (Fig. 1). All native OspC were detected, except for the one from *B. burgdorferi* strain 297. These results are in agreement with the previous C10 peptide ELISA competition experiments showing that the C10 variant peptide of strain 297 did not bind the anti-C10 peptide mAb. To evaluate the sensitivity of OspC detection by western blot, a range of recombinant OspC and whole-cell *Borrelia *in vitro culture extracts was checked. The LoD was 300 pg for recombinant OspC protein and around 1000 bacteria for all strains recognized (Fig. 1C,D).

### Detection of OspC and FlaB in a mouse model of *Borrelia* infection by western-blot

**Figure 4:**
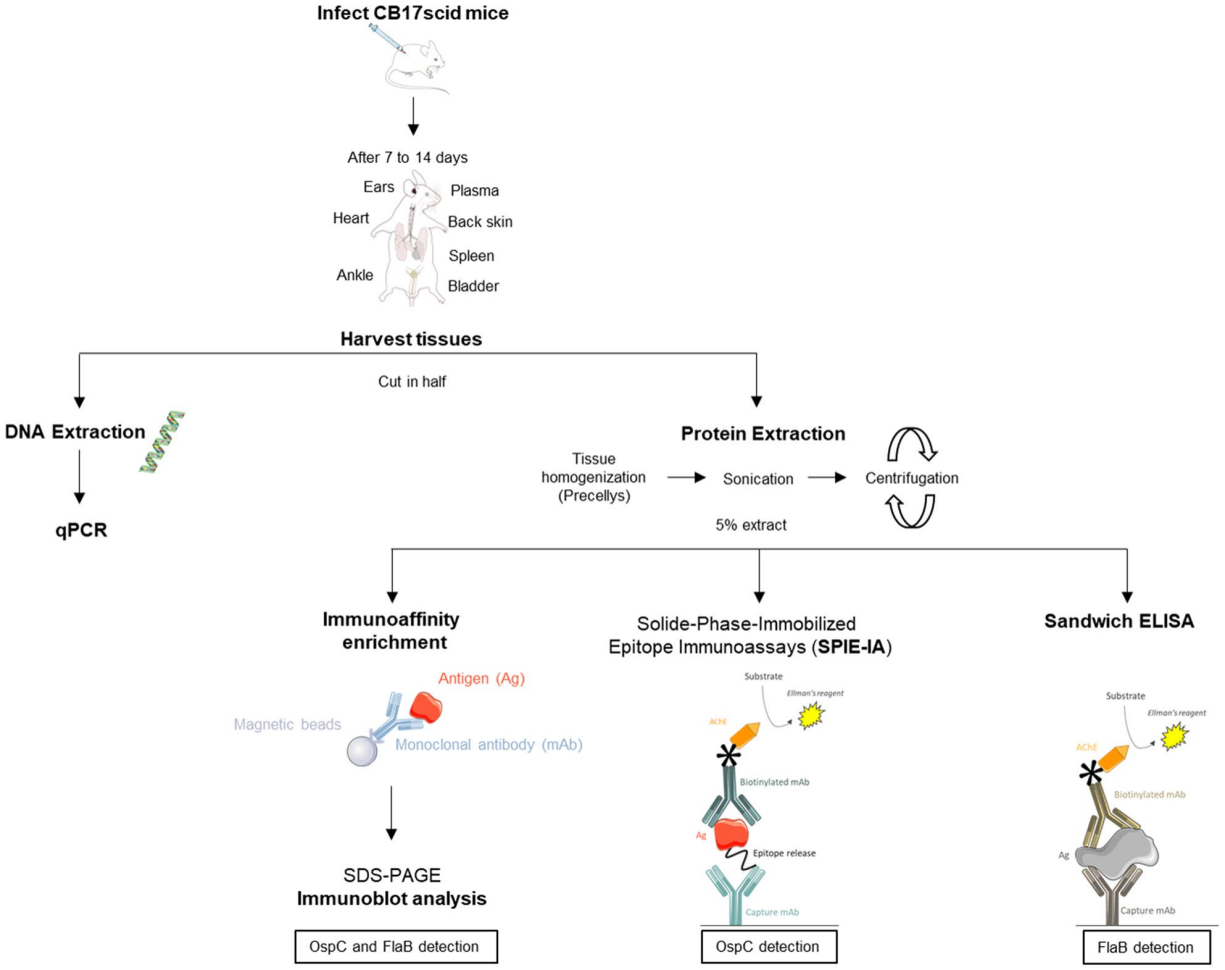
Data acquisition workflow. Schematic workflow employed for tissue analysis. After infection and sacrifice of the mice, the organs are collected for *Borrelia* quantification by qPCR and for protein analysis. Three methods are performed to detect OspC and FlaB markers: a duplex method for FlaB and OspC detection including an immuno-enrichment step before western blot analysis, a sandwich ELISA for FlaB detection, and SPIE-IA for OspC detection. This figure was created using images from Servier Medical Art (http://smart.servier.com/), licensed under a Creative Commons Attribution 3.0 License (CC BY 3.0 license: https://creative.org/licences/by/3.0/). The color of some images were modified. The mouse anatomy image is from Wikimedia Communs (https://commons.wikimedia.org), licensed under a Creative Commons Attribution-share Alike 4.0 international License (CC BY-SA 4.0 license: https://creativecommons.org/licenses/by-sa/4.0/).

**Figure 5:**
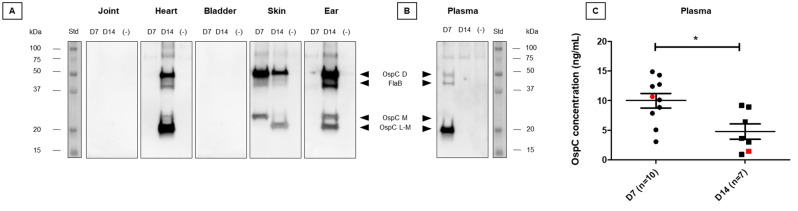
Analysis of tissue extracts from experimentally infected mice. (**A**) Western blot analysis of FlaB and OspC extracted from tissues of mice infected by the *Borrelia* BO23 strain. After immune-concentration of FlaB and OspC from tissue extracts, proteins were analyzed by western blot using a mix of biotinylated FlaB-15 and C10-11 mAbs. Data shown are representative of five mice (at 7 or 14 days). The analysis of non-infected mouse samples are included as negative controls (−). (**B**) Similarly, western blot analysis was performed with samples of plasma from infected and non-infected mice. (**C**) The graph represents the measurement by SPIE-IA of OspC in plasma of infected mice at 7 days (ten samples) and 14 days (seven samples). The statistical analysis was performed using the nonparametric Mann–Whitney U test *p < 0.05. The red dot and red square on the graph indicate plasma samples analyzed by western blot in which the amount of OspC in plasma was determined by quantitative SPIE-IA of C10 peptide. Std: Molecular weight markers and protein sizes (kDa) are indicated on the side of the gel. *D* dimer, *M* monomer, *L-M* low monomer. Uncropped original images are reported in Fig. S3.

Using our most sensitive method of detection (Fig. 4), we assayed the detection of FlaB and OspC proteins during early *Borrelia* infection in mice (Fig. 5). Mice were infected by subcutaneous injection of 10^5^ bacteria. At 7 and 14 days post-inoculation (p.i.), blood, ears, skin near the site of inoculation, heart, bladder and joints were collected and analyzed by western blot in combination with immuno-concentration. The presence of *Borrelia* in tissues was also checked by qPCR. The quantity of *Borrelia* per µg of DNA was estimated using a qPCR standard curve of *Borrelia* DNA (Supplementary Fig. S1). In control uninfected mice, no *Borrelia* DNA was detected. At 7 days p.i, 100% of skin biopsies and ears were positive, whereas other tissues were negative. At 14 days p.i, *Borrelia* DNA was detected by qPCR in skin, ears*,* heart and joints, but was not detected in the spleen or bladder. Western blot analysis of immunoconcentrated OspC and FlaB from mouse tissues at 7 and 14 days p.i showed a relatively good correlation with DNA detection. OspC and FlaB were not observed in uninfected mice. At 7 days p.i, in skin near the inoculation site, we detected three major bands around 23 kDa and 46 kDa corresponding to monomeric and dimeric forms of OspC, respectively, and a less intense band around 41 kDa corresponding to FlaB. In other tissues in which little or no *Borrelia* DNA was detected, no protein was revealed. At 14 days p.i, dimeric and monomeric OspC as well as FlaB were detected in skin, heart and ears, whereas bladder, joints, and spleen remained negative. Surprisingly, at 14 days p.i two forms of monomeric OspC were observed in ears, a 23 kDa OspC form and a smaller form around 20 kDa. In heart and skin, monomeric OspC is mainly represented by the 20 kDa form.

Even more interestingly, we can detect both OspC and FlaB in plasma. As shown in Fig. 5, at 7 days p.i a major band at 20 kDa corresponding to smaller form of OspC and two fainter bands of dimeric OspC and FlaB were detected, whereas at 14 days no protein was revealed in the plasma tested.

### Quantification of early *Borrelia* markers by enzyme immunoassay

Tissues and plasma were further analyzed by immunoassay. Given that a smaller quantity of tissue can be treated on microtiter plates, i.e. ten times less than in the immunoconcentration/western method, and that the LoD of the FlaB ELISA was estimated to be around 1 × 10^4^ sp/mL, FlaB in infected mouse samples was undetectable or unquantifiable.

Measurements of OspC in infected mouse tissues using the C10 peptide SPIE-IA are reported in Table 3. At 7 days p.i, near the site of inoculation, a very high level of OspC was measured, with an average of 400 ng/mg of skin. In distant tissues as in heart and ear, whereas a very low level of *Borrelia* DNA was detected by qPCR in some mice, up to 1.51 ng of OspC per mg of tissue was measured by immunoassay. At 14 days p.i, OspC was quantifiable in heart and ears with 98 pg–690 ng of OspC measured per mg of tissue depending on the mouse and tissue type. These results were corroborated by *Borrelia* DNA detection, but no correlation could be established between the DNA level and the very high-level OspC measured in some tissues. Possible explanations could be the heterogeneity of tissue sampling for DNA and protein samples or the heterogeneity of *Borrelia* OspC expression level in the host.

**Table 3:** Quantification of *Borrelia* and OspC in mouse tissues and plasma at the 7 and 14 days post-inoculation.

Organs	*Borrelia q*uantification (number of spirochetes/µg DNA)		OspC quantification (ng/mg tissues or ng/mL*)	
	D7	D14	D7	D14
Joint	0 ± 0 (0/5)	3 ± 6 (1/5)	0 ± 0	0 ± 0
Heart	3 ± 7 (1/5)	3478 ± 4063 (5/5)	0.45 ± 0.61	80.52 ± 172.97
Skin	1031 ± 1817 (5/5)	1916 ± 1596 (5/5)	440.15 ± 576.36	18.87 ± 36.37
Ear	6 ± 8 (2/5)	2509 ± 3071 (5/5)	0.31 ± 0.48	138.96 ± 308.08
Plasma	N.D.	N.D.	10.01 ± 3.86*	4.80 ± 3.41*

The quantity of *B. burgdorferi* in tissues was estimated by qPCR using the standard curve method (Fig. S1). The numbers in parentheses indicate the number of positive mice/number of mice tested. N.D.: not determined. OspC was measured by C10 peptide SPIE-IA. Data are presented as means ± SEM (5 infected mice per group for tissues and 10 and 7 infected mice at 7 and 14 days respectively for plasma).

Figure 5C shows the level of OspC measured in plasma at 7 and 14 days p.i. All samples (n = 17) were ELISA-positive, with a level of OspC varying from 0.5 to 14 ng/mL. Median OspC at 7 days p.i was significantly higher than at 14 days p.i (*p* value = 0.0185).

## Discussion

Currently, serological tests are recommended for the diagnosis of Lyme borreliosis^4^, although they present some serious limitations. Firstly, there is a time lag (up to 3 weeks) before detectable amounts of specific antibodies are produced by the host. Secondly, serological tests can demonstrate exposure to bacteria, but cannot prove an active infection or a reinfection^14^. Therefore, sensitive tests allowing direct detection of *Borrelia* or of components of *Borrelia* are needed. Until now, attempts using antigen capture to detect *Borrelia* antigens in blood and urine collected by non-invasive or minimally invasive sampling have not proven accurate enough and in some cases were even questionable^14^. The major difficulty in the detection of *Borrelia* in patient samples is the extremely low level of circulating bacteria and their high antigenic inter- and intra-species variability. Moreover, the antigenic repertoire of *Borrelia* changes during the course of infection, which makes the situation still more complicated.

With the aim of developing a sensitive direct diagnostic immunoassay for early Lyme disease, we focused on the detection of two potential early circulating *Borrelia* markers: FlaB and OspC. FlaB is a highly conserved structural protein of the flagella and the outer surface protein C (OspC) is a lipoprotein with high sequence variability within and between genospecies^27,28^ and is upregulated during the early phase of infection^29^. Since the capacity of an immunoassay to detect a target antigen with high sensitivity and specificity depends largely on the characteristics of the antibodies used, we decided to produce our own mAbs directed against either the FlaB protein or a highly conserved 10-amino-acid C-terminal peptide of OspC. This peptide was reported as a major immunodominant and probably surface-exposed epitope^30^, which makes it a good candidate as immunogen for production of anti-OspC antibodies. Throughout antibody screening, we paid particular attention to selection of specific and high-affinity antibodies. Of 20 anti-FlaB mAbs produced, two were selected as having the best affinity and optimal capacity to detect FlaB or different *Borrelia* strains by ELISA or immunoblot. Western blot detection was much more sensitive than ELISA in detecting *Borrelia*, with as few as ten bacteria detected per assay. For OspC, seven anti-peptide mAbs were produced, one of which, C10-11, displayed a remarkably high affinity for the recombinant OspC in the subnanomolar range.

Due to the high variability of OspC, the production of mAbs able to recognize OspC produced by all *Borrelia burgdorferi* s.l. is challenging. A least twenty five OspC major groups have been reported^27,31^ within *B. burgdorferi* s.s^32^ with up to 30% divergence in amino acid sequence between OspC groups^33^ and 58 OspC groups within the three species *B. burgdorferi, B. garinii* and *B. afzelii*^34^. It was important to consider the variability in the C-terminal OspC epitope between *Borrelia* strains and to have an overview of their impact on the binding of the C10-11 mAb. This was accomplished by performing competitive binding experiments with various peptides corresponding to the C-terminal epitope of all known full-length OspC sequences of *Borrelia burgdorferi* s.l. available in the NCBI database. Comparison of the different 10-amino-acid C-terminals of OspC shows a difference of no more than two amino acids. We showed that the C10-11 mAb was able to bind seven different peptides representing more than 90% of *Borrelia burgdorferi* strains reported in the NCBI database. This suggests that less than 10% of *Borrelia burgdorferi* s.l. could not be detected with the C10-11 mAb. To ensure their detection, production of new antibodies directed against the different C10 peptide variants are ongoing in our laboratory.

Usually, in two-site immunometric assays the antigen is taken in a sandwich between two different antibodies recognizing two distinct epitopes. In the case of OspC, to ensure the detection of a maximum number of OspC groups, we developed a SPIE-IA targeting the C-terminal epitope of OspC. In buffer or in complex mouse tissue matrices, this assay has been shown to specifically measure OspC with a LoD ranging between 17 and 100 pg/mL (i.e. around 0.1 and 0.5 fmol per assay) according to the type of sample. This is the first time that an immunoassay has been described for OspC detection. As far as we know, only one study has reported direct detection and quantification of OspC in mouse or human skin matrices, with an estimated LoD of 1 fmol of OspC peptide per assay by liquid chromatography-mass spectrometry^22^.

One of the main problems with biomarkers present in extremely small quantities in tissuesis that it is often necessary to enrich the target to make it detectable and to avoid matrix effects that can interfere with the assay. In this study, we set up an immuno-enrichment method with antibodies coupled to magnetic beads followed by western blot detection. The advantage of this method is that it is possible to analyze a larger amount of tissue than with other methods, such as immunoassays in microtiter plates, and to multiplex the detection of specific biomarkers while limiting the nonspecific signals. By combining the immuno-enrichment with anti-FlaB and anti-OspC mAbs, we were able to detect simultaneously as low as 10 bacteria (or 50 pg of recombinant FlaB protein) and 300 pg of recombinant OspC in one mL after western blot analysis in buffer or complex tissue matrices.

To evaluate the capacity of our immunological methods to detect OspC and FlaB in tissues and to investigate these two proteins as potential serum biomarkers for early human *Borrelia* infection, we used a mouse model of *Borrelia* infection after intradermal inoculation. At 7 days post-inoculation corresponding to the peak of replication at the injection site, we detected a high quantity of OspC between 7.2 ng/mg and 1.4 µg/mg of skin. These levels are consistent with our *Borrelia* DNA quantification in skin and the high level of OspC production reported in host-adapted spirochetes^35^. This is also consistent with the results of quantification of OspC by liquid chromatography-mass spectrometry in the skin of experimentally infected mice^36^, with an estimated 300 fmol of OspC (approximately equivalent to 6 ng) in 1 mg of skin biopsy. Taking into account the analytical sensitivity of western blot detection for OspC and FlaB protein, we clearly observed a higher level of OspC than FlaB in skin, ears and heart. Interestingly, in the disseminated phase we observed, in addition to monomeric and dimeric OspC, a lower molecular mass form of OspC in different tissues not found in in vitro cultivated *Borrelia*. Given that this form is recognized by the anti-C-terminal OspC mAb, it can be concluded that it corresponds to an N-terminal truncated form of OspC. There has been only one study reporting the western blot analysis of OspC protein expression in different tissues of infected mice^37^, with the detection of a unique OspC band by western blot. It is not surprising that the authors did not find the smaller form of OspC because the analysis was restricted to hydrophobic detergent-phase protein and thus to lipidated N-terminal forms of OspC.

More interestingly, we showed that it is possible to detect OspC and FlaB in the blood of infected mice. As in tissues, OspC was found to be much more predominant than FlaB in plasma. Furthermore, OspC is mainly represented by its lower molecular mass form with an apparent molecular weight of 20 kDa. At this stage of the study, we do not know if all or only a part of OspC detected in the plasma is physically bound to the bacterium. However, since OspC is anchored in the bacterial cell membrane via an N-terminal lipid, the smaller form of OspC could corresponds to circulating OspC released by bacteria. The production of *Borrelia* antigen in plasma appears to culminate on day 7 p.i as compared to day 14 p.i, with an average level of OspC around 10 ng/mL of plasma. This early detection of *Borrelia* antigen in plasma is consistent with *Borrelia* detection by culture from infected mouse blood^38^, showing a peak of positive animals between 10 and 15 days p.i. This is also consistent with the PCR detection of *Borrelia burgdorferi* DNA in blood and positive blood culture for *Borrelia* in human patients in the early phase^31,39^ or early disseminated phase^40^. As well as *Borrelia* DNA detection in blood by PCR, the detection of OspC protein in blood could be of use in diagnosis of early Lyme disease at the onset of clinical symptoms when serology testing has a poor diagnostic value.

In conclusion, this work presents the first direct detection of *Borrelia* antigen in infected animal plasma. The very sensitive immunodiagnostic tools described in this study may be of great interest for early direct diagnosis of Lyme disease using blood samples. The SPIE-IA OspC is a simple and rapid test that can be easily implemented in any clinical laboratory. It could be a useful complement to serological testing, particularly in the early phase of the disease when anti-*Borrelia* antibodies are missing. The next step would be to test a large cohort of patients with clinical symptoms to check the detectability of OspC in plasma and confirm the value of OspC as an early biomarker in blood. Furthermore, the antibodies and immune-affinity enrichment methods described in this work could be very usefully associated with methods of detection other than immunoblotting, such as mass spectrometry, to enhance their analytical performance.

## Materials and methods

### Strains and culture conditions

Low passage (fewer than five passages) *Borrelia burgdorferi* sensu lato strains (*Borrelia garinii* ATCC 51383, *Borrelia bavariensis* PBi (ATCC BAA-2496), *Borrelia burgdorferi* strain 297(ATCC 53899), *Borrelia afzelii* BO23 (ATCC 51992), and *Borrelia burgdorferi* B31 (ATCC 35210) were grown in BSK-II medium (Sigma-Aldrich, Saint-Quentin Fallavier, France) at 34 °C. The spirochetes were counted and motility was checked in a Neubauer chamber by dark-field microscopy.

### Ethics statements

All animal experiments were performed in accordance with the European Directive 210/63/ECC on the protection of animals used for scientific purposes. Animals were housed in facilities authorized by the Veterinary Inspection Department of Essonne (France) (approval numbers D911272107 and D91272106). Animal care was supervised by a dedicated veterinarian and animal technicians. Experimental procedures were conform to the European Directive 210/63/EU and were approved by the Ethics Committee of the Commissariat à l’Energie Atomique (CEtEA “Comité d’Ethique en Expérimentation Animale” No. 44) and by the French Ministry of Higher Education and Research under registration numbers APAFIS#11171-20177090615235646 v3 (for infection experiments) and APAFIS#3085-2015120909154560 v1 (for mouse immunization). All animal experiments were also performed in compliance with the ARRIVE guidelines.

### Production and purification of recombinant OspC and FlaB

The OspC gene was synthesized (GeneCust, Boynes, France) based on the published sequence in GenBank: ABA42057.1. The synthetic gene was inserted between the *NdeI* and *XhoI* restriction sites of the IPTG inducible pET22b vector, allowing the expression of OspC with an N-terminal histidine tag. *Escherichia coli* BL21 (DE3) transformed with OspC-pET22b was grown in Luria broth with 100 µg/mL ampicillin at 220 rpm and 37 °C until the mid-log phase was reached (OD at 600 nm = 0.6) and induced with the addition of 1 mM isopropyl-β-d-thiogalactoside (IPTG) for 4 h at 37 °C. The culture was centrifuged for 20 min at 2500×*g* at 4 °C and cells were resuspended in binding buffer A (50 mM potassium phosphate buffer pH 7.4, 300 mM NaCl) supplemented with 1 mM 4-(2-aminoethyl) benzene sulfonyl fluoride hydrochloride. The cells were disrupted by sonication (1 min at 14 W) and then centrifuged at 14,000×*g* for 20 min at 4 °C. The final supernatant was purified on pre-equilibrated nickel chelating Sepharose Fast flow (Cytiva, Vélizy-Villacoublay, France). After washing with binding buffer, elution of recombinant OspC protein was performed with binding buffer A supplemented with 0.5 M imidazole. The eluted fractions were pooled and dialyzed against 50 mM potassium phosphate buffer, pH 7.4, 150 mM NaCl. Purified OspC recombinant protein was quantified using the Pierce BCA protein assay kit (Thermo Fisher Scientific, Illkirch, France) and the purity was assayed by electrophoresis using the Protein 230 kit and the Agilent 2100 Bioanalyzer system. OspC was stored at − 20 °C until use. Recombinant FlaB (synthetic gene based on the sequence published in GenBank: GU826786) was produced as described above for OspC, except that the binding buffer A was replaced by a denaturing buffer containing 100 mM potassium phosphate buffer, 10 mM Tris, 150 mM NaCl, 8 M urea, pH 8.

### Production of monoclonal antibodies (mAbs)

Four 12-week-old female *Biozzi* mice (bred in the animal care unit at CEA) were immunized by four intraperitoneal injections at 3-week intervals of 50 µg of FlaB protein mixed with alum adjuvant or 50 µg of C10 peptide: H-CPVVAESPKKP-O conjugated to bovine serum albumin (BSA)-succinimidyl-4-(*N*-maleimidomethyl) mixed with alum adjuvant. The two mice presenting the highest antibody titer (see “ELISA method” below) were selected and received three intravenous injections of 50 µg of FlaB or C10-BSA. Two days after the last boost, hybridomas were produced by fusing spleen cells with NS1 myeloma cells and were subsequently cloned by limiting dilution as previously described^41^. Throughout the screening process and cloning steps, hybridoma culture supernatants were tested for the presence of anti-FlaB antibodies by ELISA. mAbs were produced in culture supernatant and purified by protein G affinity chromatography (HiTrap Protein G; GE Healthcare, Buc, France) using an AKTAxpress system (Cytiva, Velizy Villacoublay, France).

### Protein and peptide labeling

mAbs and recombinant protein were labeled with biotin for use as conjugates. Briefly, 0.67 nmol of antibody dissolved in 400 µL of 0.1 M borate buffer pH 9 was reacted with 13.3 nmoles of biotin-N-hydroxysuccinimide ester (Sigma-Aldrich, Saint-Quentin Fallavier, France) dissolved in water-free DMF. After a 1-h reaction at room temperature (RT), 100 μL of 1 M Tris–HCl pH 8.0 was added for 1 h at RT. Finally, 500 μL of enzyme immunoassay (EIA) buffer (see “Composition” below) was added and this preparation was stored at − 20 °C until use. OspC10-peptide was conjugated to the tetrameric AChE enzyme (G4) as previously described^42^. Briefly, 2 nmol of C10 peptide-SH was reacted with 0.1 nmol of AChE-SMCC (maleido group inserted into AChE) in sodium phosphate buffer pH 6, 5 mM EDTA overnight at 4 °C. The tracer C10 peptide-AChE was then purified by gel filtration on a Zeba spin desalting column 7 K MWCO (Thermo Fisher Scientific, Illkirch, France).

### Enzyme immunoassays

To titrate anti-FlaB and anti-C10 peptide antibodies in mouse sera and in hybridoma culture supernatants, microtiter plates (MaxiSorp, Nunc) coated with goat anti-mouse antibodies (Jackson ImmunoResearch, Cambridgeshire, United Kingdom) at 5 µg/mL and blocked with EIA buffer (0.1 M potassium phosphate buffer pH 7.4, containing 0.1% BSA, 0.15 M NaCl and 0.01% sodium azide) were prepared. Before use, plates were washed with washing buffer (10 mM potassium phosphate buffer pH 7.4 and 0.05% Tween 20) and then 50 µL of serum dilution or hybridoma culture supernatant was loaded per well and reacted for 2 h. After three washes, plates were reacted with 50 μL per well of 100 ng/mL of biotinylated FlaB or 1 Ellman unit (EU)/mL of AChE-labeled C10 peptide. For FlaB, plates were washed again and reacted with AChE-labeled streptavidin for 30 min^43^. After three washes, AChE activity was revealed by addition of Ellman's reagent^44^. For evaluation of the recognition of *Borrelia* by the antibodies, we used a direct ELISA. For this purpose, 96-well microplates (MaxiSorp, Nunc) were coated with 100 µL per well of sonicated *Borrelia afzelii* BO23 at 10^5^ sp/mL and then blocked with EIA buffer (0.1 M potassium phosphate buffer pH 7.4, containing 0.1% bovine serum albumin (BSA), 0.15 M NaCl and 0.01% sodium azide) for at least 4 h at RT. Then 50 µL of serum dilution or hybridoma culture supernatant was loaded per well and reacted for 2 h at RT. After three washes, plates were reacted for 2 h at RT with 100 μL per well of 1 EU/mL of AChE-labeled goat anti-mouse antibodies. After three washes, AChE activity was revealed by addition of Ellman's reagent.

### SDS-PAGE and immunoblot analysis

For western blotting, bacterial suspensions of different *Borrelia* strains or recombinant OspC and FlaB were suspended in Laemmli buffer containing 2% SDS and 2-mercaptoethnol, denatured for 5 min at 95 °C, and run on SDS PAGE (10 or 13.4% resolving). Proteins were transferred onto a polyvinylidene difluoride membrane using the Trans-Blot turbo Transfer System (Bio-Rad, Marnes-la-Coquette, France). Briefly, the membrane was blocked with 5% BSA in phosphate buffer saline containing 0.1% Tween 20 (PBST). Each mAb labeled with biotin was diluted to 0.5 µg/mL in 1% BSA in PBST and reacted for 1 h at RT with the proteins transferred to the membrane. The membrane was subjected to three washes in PBST and incubated for 20 min at RT with 1:40,000 Pierce Streptavidin Poly-Horseradish Peroxidase (Thermo Fisher Scientific, Illkirch, France) in 3% BSA in PBST. After four washes in PBST, the proteins were detected by chemiluminescence (ECL Prime, GE Healthcare, Buc, France), using the ChemiDoc Imaging System (Bio-Rad, Marnes-la-Coquette, France). Western blots were analyzed using the ChemiDoc Touch Imaging System. Images are processed with Image Lab Touch Software version 2.2.0.08. All exposures used correspond to the optimal exposure of the system with optimal settings regarding resolution wait time.

### Combinatorial analysis

To evaluate the best mAb pairs to be used in a two-site immunometric test for FlaB detection, a combinatorial analysis was carried out using each mAb either as capture or conjugate antibody using FlaB or a suspension of sonicated *Borrelia burgdorferi* as a target. Immobilization of the capture mAb in microtiter plates was performed by distributing 100 µL/well of the antibody at a concentration of 10 µg/mL in 0.05 M potassium phosphate buffer pH 7.4 and incubating the plates overnight at RT. The plates were then saturated with EIA buffer for 4 h at RT, and stored at 4 °C until use. Plates were then washed three times and 100 µL of a suspension containing 10^6^ spirochetes/mL of sonicated *Borrelia afzelii* or 100 µL of a recombinant FlaB (10 ng/mL and 1 ng/mL) was loaded per well. After overnight incubation at 4 °C and three washing cycles, 100 µL of biotin-labeled tracer mAb (100 ng/mL) was added and reacted at RT for 4 h. Plates were washed, and after addition of 100 µL/well of AChE-labeled streptavidin conjugate (2 EU/mL) were reacted at RT for 1 h. After three washes, AChE activity was revealed by Ellman's colorimetric method. A similar combinatorial analysis was performed for the selection of the best anti-C10 peptide antibody pairs using recombinant OspC (from 100 pg to 2 ng/mL) and the SPIE-IA method.

### Solid phase-immobilized epitope immunoassay (SPIE-IA) for OspC detection

The SPIE-IA method developed several years ago in our laboratory^23^ allows the measurement of small molecules in a “two-site immunometric” format. This method is based on the use of a single epitope recognized successively by the capture antibody and the tracer antibody, and involves covalent cross-linking of the analyte to the solid phase.

Briefly, OspC samples were reacted with solid phase (96-well microplates; MaxiSorp, Nunc) coated with anti-C10-peptide mAbs for 2 h at RT. After a first washing step, the solid phase-bound OspC was cross-linked with the capture mAb using glutaraldehyde (0.1% for 5 min at RT) before a second washing step. Residual glutaraldehyde reactivity was neutralized and solid phase-bound proteins were denatured with borate trimethylamine (10 mg/mL) in methanol/HCL (v/v). After a third washing step, biotinylated C10-11 mAbs were added at 100 ng/mL and reacted for 2 h at RT. After a new washing step, solid phase-bound OspC was reacted with AChE-labeled streptavidin conjugate (home-made, 1 EU/mL) and then revealed with Ellman’s reagent.

### Biolayer interferometry

Antibody binding kinetics were measured using biolayer interferometry on an Octet Red96e from fortéBio (Sartorius, Fermont, CA, USA) according to the manufacturer’s instructions. Briefly, anti-FlaB or anti-C10 peptide mAbs were loaded onto anti-mouse IgG Fc-capture (AMC) biosensors from fortéBio (Sartorius, Fremont, CA, USA) previously hydrated by immersion in an assay buffer (EIA 0.02% Tween20) for at least 10 min. Assays were performed with shaking at 1000 rpm and at 25 °C. For K_D_ determination, each mAb was loaded onto the sensor at 10 µg/mL, following by a baseline step in EIA 0.02% Tween20 buffer. The sensors were exposed to different Flab or OspC concentrations (from 200 to 5 nM) and association was measured for 300 s, followed by 10 min of dissociation in buffer. The sensor surfaces were regenerated by dipping in 10 mM glycine buffer at pH 1.3 for 20 s. Data analysis was performed using the Octet Data analysis software, version 10.0 (fortéBio LLC, Sartorius, Fremont, CA, USA). After reference sensor subtraction (sensor dipped in 0 nM of antigen), experimental data were fitted with the binding equations describing a 1:1 binding model. The association rate constant (k_a_), dissociation rate constant (k_dis_), and equilibrium dissociation constant (K_D_) were obtained. The K_D_ values were calculated as the ratio k_dis_/k_a_.

### Competitive ELISA

The apparent affinity of anti-C10 peptide mAbs for different variants of C10 peptides and recombinant OspC was measured by competitive ELISA, using goat anti-mouse antibodies (Jackson ImmunoResearch, Cambridgeshire, United Kingdom) coated on the wells of a microtiter plate. A mix of anti-C10 peptide mAb (1 ng/mL), AChE-labeled C10 peptide (0.5 EU/mL) and different concentrations of competitor peptide or recombinant OspC were reacted overnight in the wells at 4 °C. After three washes, AChE-bound activity was revealed with Ellman’s reagent for 1 h. The peptides synthesized by Genecust were > 85% pure: PVVAESPKKP, PVVAEAPKKP, PVVAESPKKT, PVVAESPKNP, PVVAETPKKP, PVVAENPKKP, PVVVESPKKP, PVMAESPKKP, PVVAKNPKKP, PVVAKSPKKP, PVVAKTPKKP, PIVAESPKNP, PIVAESPKK.

### Infection of mice with *Borrelia burgdorferi* s.l.

Four-week-old female CB17scid mice from Janvier were subcutaneously infected with 10^5^ *Borrelia afzelii* BO23 strain in BSK medium. In parallel, a negative control group (n = 5) was injected with BSK medium. At 7 or 14 days post-inoculation mice (five mice per group at different times after infection) were sacrificed and the blood, heart, bladder, joints, skin at the inoculation site and ears were collected for DNA amplification and immunoassays. Two more mice were included in the study for plasma analysis at 14 days post-inoculation.

### *B. burgdorferi* DNA detection in mouse tissues

Mouse DNA extraction was performed using the DNeasy Blood and Tissue Kit (Qiagen, Hilden, Germany). The purified DNA was eluted with AE buffer (10 mM Tris-Cl, 0.5 mM EDTA pH 9) in 200 μL for each mouse organ and tissue. PCR was performed on each sample to amplify the 5S-23S intergenic spacer region. Amplification was performed in a CFX96 Touch Real-Time PCR Detection System (Bio-Rad, Marnes-la-Coquette, France) with a 20 μL final volume containing 10 µL Fast EvaGreen master mix, 400 µg of BSA/μL, 500 nM of each primer RRC (5′-CTGCGAGTTCGCGGGAGAG-3′) and RRB (5′-AAGCTCCTAGGCATTCACCATA-3′)^45^ and 2 µL of DNA samples. The PCR cycles were carried out with an initial denaturation for 3 min at 95 °C; 40 cycles of denaturation for 10 s at 95 °C, annealing for 30 s at 63.4 °C. After amplification, a melting curve was acquired by heating the product from 65 to 95 °C for 5 min. Melting curves were used to determine the specificity of the PCR. To determine the copy number of the target genes in the samples, a standard curve was established with known amounts of purified *Borrelia* DNA. The matrix effect of mouse DNA on the amplification of *Borrelia* DNA was checked and had no impact on the Cq values.

### Sample preparation—protein extraction

Mouse tissues were collected in a clean tube with aseptic instruments and stored at − 20 °C until use. Tissue samples (15 mg) were placed in homogenizer tubes with two ceramic matrix CK28 (Bertin Technologies, Montigny-le-Bretonneux, France) and 300 µL of lysis buffer (50 mM Tris pH 8, 150 mM NaCl, 1% IGEPAL, 0.5% sodium deoxycholate) supplemented with protease inhibitor cocktails (Sigma-Aldrich, Saint-Quentin Fallavier, France). Samples were processed with a Precellys 24 tissue homogenizer (Bertin Technologies, Montigny-le-Bretonneux, France) using four cycles of 20 s at 6500 rpm. Then, samples were sonicated twice for 1 min. Samples were centrifuged for 10 min at 15,000×*g*, supernatants were collected and protein concentrations were determined using the Pierce BCA protein assay kit (Thermo Fisher Scientific, Illkirch, France).

### Immunocapture of OspC and FlaB combined with western blot analysis

To improve the detection of both FlaB and OspC antigen in tissues and serum samples, we developed a concentration method based on immunoaffinity enrichment on magnetic beads (Dynabeads M-280 tosyl-activated, Thermo Fisher Scientific, Illkirch, France) followed by western blot detection. First, we optimized tissue extraction by testing different extraction buffers and mechanical cell disruption conditions. The efficiency of extraction was checked by protein quantification (see “Methods”).

The effectiveness of immunoconcentration experiments is an important factor for detection of low-abundance proteins, especially in complex matrices. To determine the optimal conditions for immunocapture of OspC and FlaB, we investigated different parameters, including (1) selection of antibodies bound to beads, (2) sample dilution, (3) time of immunocapture, and (4) washing step. The efficiency of immunocapture using the best conditions i.e. immunocapture of 0.5% tissue extract (W/V) or tenfold diluted plasma with FlaB15 and C10-11 mAbs for 1 h, three washes and elution in Laemmli buffer yielded over 90% of FlaB and OspC from 5 mg of tissues or 50 µL of plasma.

Finally, Immunoprecipitation was performed as follows: mAbs (200 µg) were coupled to 10 mg of magnetic beads and saturated with BSA as recommended by the manufacturer. Samples (100 µL) were diluted tenfold with EIA buffer and incubated with 20 µL. of beads coupled to antibodies for 1 h at RT. After three washes with PBS-Tween, the beads were resuspended in 15 µL of Laemmli buffer (without 2-mercaptoethanol). Incubation and washing steps were performed on a King Fisher Duo Prime robot (Thermo Fisher Scientific, Illkirch, France) and bound protein was analyzed by immunoblot.

### Theoretical limits of detection (LoD) and quantification (LoQ) of immunoassays

For all immunoassay formats, LoD and LoQ were calculated using PRISM^®^ software v.5.04 (GraphPad Software Inc., San Diego, CA, USA) with a nonlinear regression model using a two-site specific binding curve fit. LoD is defined as the lowest protein concentration giving a signal greater than the nonspecific binding calculated as the mean of 12 measurements of the buffer or unspiked matrix + 3 standard deviations. LoQ is defined as the lowest concentration of protein that can be quantitatively determined and is calculated as the mean of 12 measurements of the buffer or unspiked matrix + 10 standard deviations.

## Supplementary Information

Supplementary Figures.
