# Supplementary material for: Detection of Borrelia burgdorferi antigens in tissues and plasma during early infection in a mouse model
Source: Sci Rep. 2021 Aug 30;11:17368. doi: 10.1038/s41598-021-96861-z (PMC8405660; doi:10.1038/s41598-021-96861-z)
Supplement: Supplementary file 1 — Supplementary Figures. [file 41598_2021_96861_MOESM1_ESM.docx]

**Supplementary data**

**Detection of *Borrelia burgdorferi* antigens in tissues and plasma during early infection in a mouse model**

**Victoria Dolange^1^, Stéphanie Simon^1^ and Nathalie Morel^1^**

Supplementary Figure S1

**Standard curve of qPCR using serial dilutions of *B. afzelii* BO23 strain DNA.**


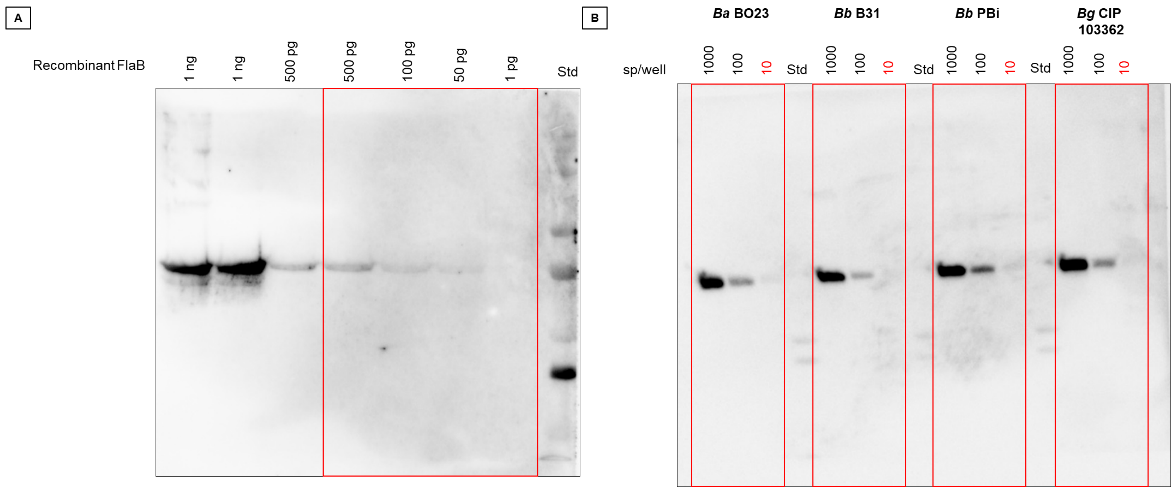


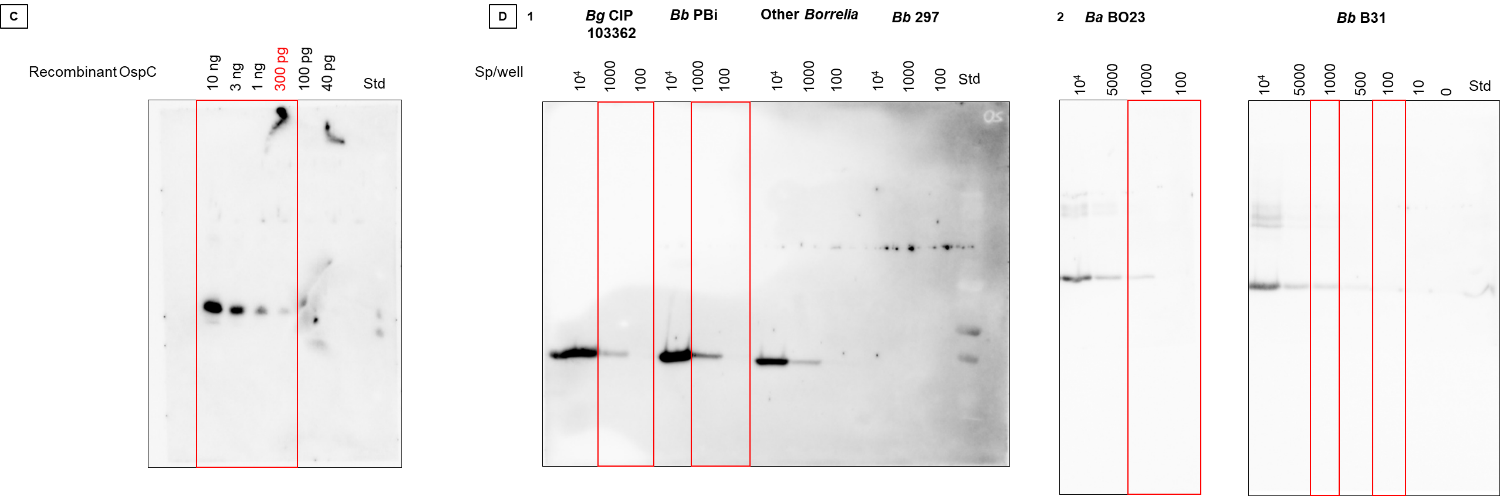


Supplementary Figure S2

**Original, uncropped images of western blot membranes shown in Figure 1. [A]** Exposure: Auto optimal 395.082 s **[B]** Exposure: Auto optimal 28.999s **[C]** Exposure: Auto optimal 7.523 s **[D] 1.**Exposure: Auto optimal 64.465 s **2.**Exposure: Auto optimal 24.333s. Red boxes represent the regions used in the main figures. The standards correspond to the Chemidoc chemiluminescence image, for a better visibility of the markers, the images were also captured by colorimetry.


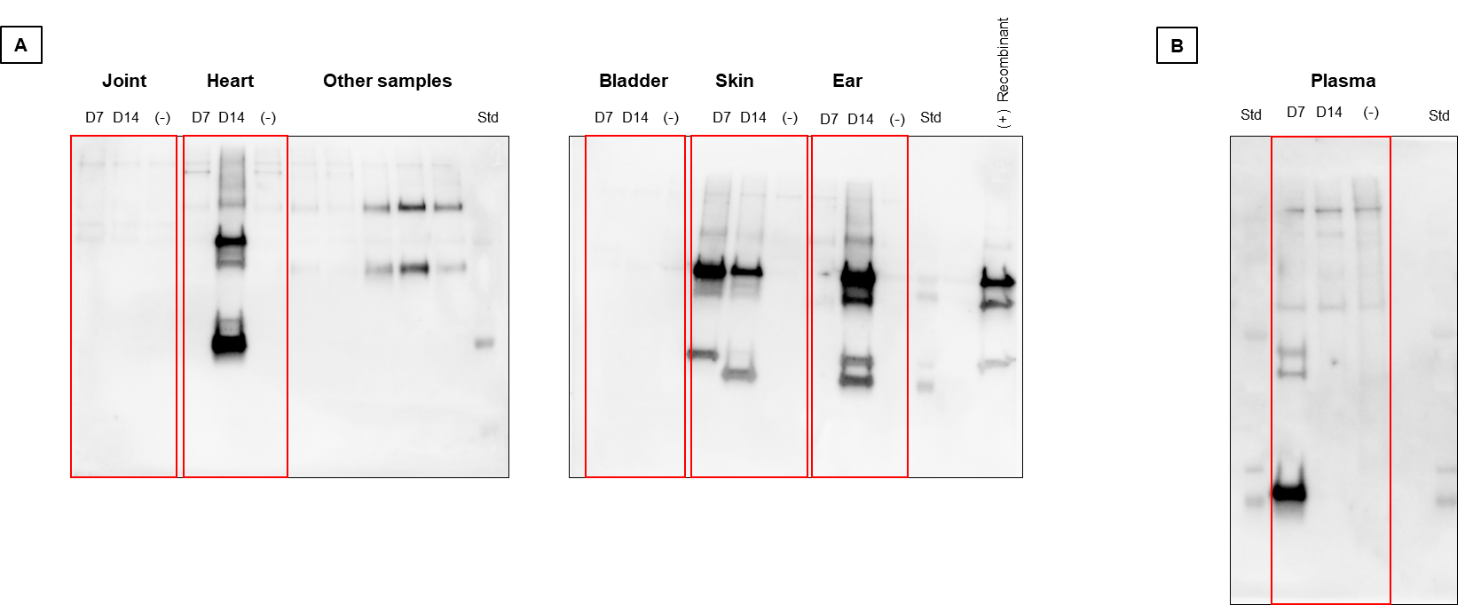


Supplementary Figure S3

**Original, uncropped images of western blot membranes shown in Figure 5. [A]** Exposure: Auto optimal 33.629 s **[B]** Exposure: Auto optimal 53.752 s. Red boxes represent the regions used in the main figures. The standards correspond to the Chemidoc chemiluminescence image, for a better visibility of the markers, the images were also captured by colorimetry.
